# Research capacity building through North–South–South networking: towards true partnership? An exploratory study of a network for scientific support in the field of sexual and reproductive health

**DOI:** 10.1186/s12961-017-0202-z

**Published:** 2017-05-05

**Authors:** K. Van der Veken, L. Belaid, T. Delvaux, V. De Brouwere

**Affiliations:** 1 Woman and Child Health Research Centre, Department of Public Health, Institute of Tropical Medicine, Antwerp, Belgium; 2 Centre de Recherche du Centre Hospitalier de l’Université de Montréal (CRCHUM), École de Santé Publique de l’Université de Montréal, Montreal, Canada

**Keywords:** Research networks, North-south, South-south, Capacity strengthening, Low- and middle-income countries, Qualitative research, Reproductive and sexual health

## Abstract

**Background:**

We explored the perceptions of members of the Network for Scientific Support in the field of Sexual and Reproductive Health (NetSRH) on North–South–South networking and on constraints and perspectives for South-led research.

**Methods:**

An exploratory qualitative study was conducted 18 months after the network was launched. In-depth interviews were carried out with NetSRH members (n = 15) affiliated to southern research institutions. A thematic analysis was done and N-Vivo 10 software used.

**Results:**

A number of barriers to South-led research were identified, the most important being a lack of time, resources and research skills, and donor influence for the choice of research topics. Although the level of technical skills, such as writing proposals and scientific papers, differed among NetSRH members, all welcomed additional research capacity building. All members have deplored the lack of research management skills such as project cycle management as well as how to communicate with and get funds from donor agencies. International (local or regional) donor agencies had their own agenda with a budget already reserved for other purposes, thus priorities identified by national researchers were less taken into consideration. Systemic dependencies on external funds lead southern research partners to respond to calls for proposals mostly initiated by partners from northern institutions, leaving limited leeway for local initiatives. Southern NetSRH members perceived coaching done by the northern partners in scientific writing positively. South–South collaboration was minimal within NetSRH at this stage of the project, mainly due to time and resources constraints.

**Conclusion:**

NetSRH members unanimously concluded that sustainable financing of southern research centres is a necessary condition for them to initiate their own research projects. We recommend reserving funds within the international donor agencies for South-led research in order to break the vicious circle of running behind money provided by northern donors, thereby missing out on time and resources for reviewing research gaps and/or conducting needs evaluations required to initiate relevant own research.

## Background

In order to reinforce the health system and to improve the health of populations, strengthening of research capacities is fundamental [1–3]. Research capacity strengthening refers to a continuous process with the aim to strengthen the sovereignty of individuals, institutions, organisations and nations in defining and prioritising health problems, in developing and evaluating in a scientific manner the appropriate solutions to the identified problems, and in applying and sharing the knowledge generated by the research [1].

The promotion of research activities by northern countries in low- and middle-income countries (LMICs) has known different modalities and evolved from problem solving over technical assistance with a capacity-building component, to collaborative partnerships [4]. The creation of effective research systems indeed depends not only on human and financial resources but also on the professional and social status of research personnel, adequate reward systems and the emergence of new collaborative relationships [4]. North–South collaboration is an opportunity to bring in funds, expertise and resources to conduct research in low-income countries. Nevertheless, it may also generate perverse effects in reinforcing the power asymmetry between northern and southern institutions. Chu et al. [5] and Barrett et al. [6], among others, have demonstrated that, in the past, northern institutions have been criticised for conducting research in southern countries without fair collaboration with the local partners. We notice that partners in LMICs implement mainly research projects designed and financed by northern partners [7, 8], including consultancies for United Nations (UN) agencies and international non-governmental organisations. Simultaneously, in many African countries, we observe a weak national health research system [9]. In West Africa, for example, the budget for health research represents only 0.5% of the national budget and the budget allocated to health represents only 1% of the net domestic product [1].

It is in a perspective of readjustment of partnership that, since 1998, the Institute of Tropical Medicine (ITM) in Antwerp, Belgium, has been running an extensive program on sustainable scientific, medical and veterinary capacity building in the South with support from the Belgian Directorate-General for Development. The program aimed to strengthen both the individual and institutional research capacity of southern partners so that “*partner institutes gradually would take charge of developing scientific and medical expertise, as they own, lead and are held accountable for their role in the partnership*” ([10] p. 1). One of the networks born from this program in January 2014 was the Network for Scientific Support in the field of Sexual and Reproductive Health (NetSRH) [11], which brought together research centres from five West African and three North African countries.^1^ The purpose of this 3-year project was to develop and strengthen a network of partners in North and West Africa conducting (collaborative) research that can be translated into policy and practice.

After 18 months of the NetSRH project it became manifest that initiating and leading proper research is not without constraints for NetSRH members. Therefore, the ITM set up an exploratory study in order to better understand these constraints, attempt to lift them and support their partners in a tailor-made manner, i.e. according to their needs, during the second half of the project.

The objectives of our study were to document obstacles for southern research institutions in reinforcing their research capacities and to understand the perceptions of southern researchers on capacity transfer through North–South and South–South collaboration. The results of this study may contribute to the design of interventions aimed at strengthening the capacity of southern researchers and to improving research collaboration modalities.

## Methods

### Setting: some background on NetSRH

The objective of this project was to develop and reinforce a network of partners in North Africa (Morocco, Algeria, Tunisia) and West Africa (Burkina Faso, Guinea, Ivory Coast, Senegal and Benin) to reinforce their research capacity in the field of sexual and reproductive health (SRH) and to produce scientific research that can be translated into policies and practices. One of the selection criteria for research questions was to respond to the needs of the country.

In this project, the principal role of the northern institution (ITM) was to accompany and coach the research teams in the development of a SRH research protocol up until its successful submission to a donor, its implementation and, if possible, the analyses of the collected data. NetSRH foresaw as a result the initiation of a dialogue on the research topic studied within NetSRH with policy or program actors at national levels (and possibly influencing national health policies and programs through preliminary results of the study). Considering the short period of the pilot project (3 years), the publication in international journals was not included in the deliverables.

Twenty researchers, i.e. two per southern institution (n = 16) and four from the ITM were continuously involved in the network since its commencement. Institutions were identified and selected by the ITM on the basis of earlier collaborative experiences and their researchers’ expertise in one of four main topics regarding SRH, namely family planning, breast cancer, quality of maternal healthcare and prevention of HIV for vulnerable groups. The network members met annually in a 4–5 days network meeting, where the process and intermediate results of the project were discussed and capacity-building sessions organised around themes that the network members identified as important. In 2014, the kick-off meeting took place in Morocco, with an introduction on sex- and rights-based research and capacity building on literature review and grant writing. The focus was on identification of research topics and study designs. The second meeting took place in 2015 in Senegal and concentrated on peer-review of research proposals, capacity strengthening in qualitative research and ethics, and identification of donors for research funding. The final meeting (held after this study was conducted) took place in 2016 at the ITM, Belgium, and focused on further training concerning grant writing, qualitative data analysis and policy change through, among others, the use of policy briefs. More information on the activities of the NetSRH project can be found elsewhere [11].

### Study design, data collection and analysis

Between June and December 2015, i.e. 18–24 months after the NetSRH was launched, an exploratory qualitative study was carried out within the network project. The population of the study consisted of all members of the SRH network. We used a purposive sampling strategy [12]. The sample was diverse in itself in terms of sex, position, professional affiliations, seniority and experience in research. Seven of 15 researchers interviewed were female. All of them were trained in quantitative research but only few had in-depth knowledge of qualitative research. Half of the researchers were occupying a senior position and dealt with research management.

In-depth interviews were conducted with 15 southern researchers, two from each southern NetSRH country member, except for one member country in which only one researcher was available for the interview. At the time of data collection, the interviewer was an ITM researcher and part of the NetSRH management team. After having collected the data, a second researcher, new at ITM and in the NetSRH management team, analysed the data. The potential bias caused by the ITM being not only a partner in NetSRH but also responsible for the administrative management, and by the authors of this article being exclusively ITM researchers, is the reason for not interviewing northern (ITM) researchers. The interviews were held through Skype from the ITM office and recorded (Skype recorder). Partners from the same institute were interviewed together. An interview guide was used exploring the themes of workload, time and resource availability to focus on research, the selection process of the research topic, and difficulties encountered during proposal writing and submission to a donor. Additionally, perceptions on research capacity building and on North–South and South–South collaboration in general and as experienced within NetSRH were inquired. The average length of the interviews was 1 hour. Data collected from interviews were audiotaped and transcribed in French. Data were organised and reduced using the qualitative data management tool N-Vivo 10. Based on hypotheses drawn from the literature [2], a thematic analysis was conducted using a mixed approach (deductive and inductive approach) [13] as can be seen in Box 1. This has been an iterative process.

Box 1: The structure used for thematic analysis

**Table:** 

Category/nodes/sub-nodes. *Italic = hypotheses drawn from literature* [5]	
Perceived obstacles to South-led research	
Proper identification of a research theme	
Limited resource availability	
	*Human:* required competences or knowledge not available
	*Financial:* no initial budget for research proposal development
	*Internet and communication:* access to information
	*Time for research*: prioritisation of financially rewarding projects
*External funding constraints:* differences in agenda and topic prioritisation; availability of budget	
Specific experience with networks or partnerships	
Perceptions of southern NetSRH members on network collaboration between South and North	
The appreciation of North–South collaboration	
Challenges and successes of NetSRH	
Reasons for limited South–South collaboration	
	Perceived need for collaboration
	Communication
	Financial perspectives
	Contextual differences
Role for North in North–South network	

#### Ethical considerations

The Institutional Review Board of the ITM has approved the research (No. 1005/15). An informed consent was given by email and confirmed during the interview. It was specified that the names of participants would not appear in any document. The transcribed interviews and audiotapes were coded and only the research team had access to the list of names and codes. No participant can be identified in the publication of study results.

Our study has several limitations, one clearly being the fact that it has been conceived, performed, and analysed by the (sole) northern partner within NetSRH, hence the likelihood of a response bias. Respondents may feel dependent on the northern partner, which is the administrative manager of NetSRH, and therefore not feel free to criticise the way ITM has managed the budget. The authors have tried to limit this bias by reassuring members that there is no impact of this study on their integration in our network and by confirming that the objective of this study is to better understand their constraints in order to be able to improve South–North/South collaboration and to enhance opportunities for South-led research. NetSRH members read the manuscript and agreed upon the results to enhance the internal validity of the findings. Because our study is an exploratory study based on the experience of one research network we cannot extrapolate nor generalise results to all North–South research networks, thus limiting the external validity of the results.

## Results

In the following section we discuss the challenges to implement South-led research as perceived by NetSRH members and their perceptions on North–South and South–South partnerships. The results are organised according to the structure of the thematic tree used for analysing the results (Box 1).

### Perceived challenges to implement South-led research

#### Limited resource availability

##### Human resources

All respondents reported being in favour of more research capacity building. While the majority of NetSRH members were trained and experienced in quantitative research, fewer were familiar with qualitative or mixed methods. A number of research topics identified by the NetSRH members at the kick-off meeting required a more in-depth understanding of users or community perceptions and barriers, for instance, the use long-term family planning methods. Challenges encountered during the writing of research protocols concerned the parts related to research methods and ethical issues but also to writing scientific papers and to research management skills such as grant writing and communication with donors. NetSRH members felt these management skills crucial for successfully implementing research projects, and were lacking among southern researchers in comparison to northern partners: “*It requires a particular competency to set a common work agenda. To stick to the plan, to update the information, to remind everyone involved of his or her responsibilities*” (R13). With regards to research management skills, one member formulated the differences as follows: “*When you ask an institute of the south to create a project with a logical framework, with indicators, with outputs, deliverables, milestones, people are lost. They are used to writing narratives*” (R11)*.* Finally, knowing how to publish was seen as an important advantage of the North over the South: “*We are not used to publish, we need to be guided*” (R3). Since all NetSRH members are French speakers, challenges linked to publication might also be rooted in a lack of English writing skills.

##### Financial resources

The fact that NetSRH had no allocated funds available for implementing the elaborated research proposals of its members was considered a major constraint for conducting research. Conducting relevant country- or regional-based research requires literature reviews and/or needs assessments to enrich a research proposal, which are time and resource consuming activities. “*To prepare local projects, you need a local budget, and if you don’t have the local resources to finance the preliminary phase it becomes complicated.* […] *Initiating its own research projects would be ideal but requires investment*” (R11). Because of the lack of own funds and financial reserves, southern researchers depended to a large extent on external donors. Challenges concerning the search for funds were said to be related to a specific donor architecture and deserved so much attention in NetSRH members’ responses that they are mentioned under a separate heading below.

##### Internet and communication means

NetSRH members reported access to information concerning calls for research as a challenge. Accessing funds is only possible through access to information and systematic follow-up of calls for proposals in order to prepare a timely response. Problems of communication and internet connection were mentioned by nearly all respondents, constraining some of them in elaborating a research proposal in a timely manner. In addition, all NetSRH members were French-speaking, therefore access to information on grants as well as on technical matters was more difficult. “*I think the language equally represents a barrier for many of the resources are available in English. If we were more open to this language, I think that would facilitate things*” (R15)*.*

##### Time for research

NetSRH’s members estimated the time available for research ranging from 20% to 65%. In addition to ongoing research within their institution, NetSRH members were engaged in multiple activities, for example, teaching and coaching activities, clinical work and management, including administrative tasks and budgetary follow-up. This multiple engagement has negatively affected the participant’s availability for research activities within the network. Three NetSRH research partners were attached to, or working in close collaboration with, the Ministry of Health, which caused a considerable amount of time to be spent on the collection and analysis of epidemiologic and Health Management Information System data. Respondents from all five West African research centres also stated that they spent most of the time available for research by responding to calls, mostly by northern institutions, in order to make ends meet. This financial pressure represents a day-to-day constraint in initiating South-led research. “*We are submerged by the daily work*” (R4). “*All have the same problem they have to struggle to survive*” (R8)*.*

#### External funding constraints

Why is it that South-led research projects do not easily get financed? “*Governmental structures in terms of research regulation and research funding are absent in our country.* […] *The national research institutes are abandoned*” (R12)*.* In search of funds, several NetSRH members mentioned the importance of networking, an activity for which they state not having time. Some mention “*a heavy procedure*” (R3) due to the fact that central authorities have made an agreement with the major external donors, which leaves little leeway for other actors: “*We do not have the liberty to receive a grant, whether it is from a public or private donor. We work with NGOs and it is they who receive the budget*” (R3)*.*

South-led research seems hampered by the actual donor architecture from the start, research topics being decided upon in function of the potential interest of in-country donor agencies rather than in function of local needs. NetSRH members approached in-country donors at an early stage (before the fifteenth month, according to the project’s objectives) to share research ideas and to investigate their willingness to fund them. Two main reasons were given by respondents for the approached donors, such as UN agencies, to not fund NetSRH research proposals. First, donors and research institutes often had different agendas. Donors had funds available for interventions and operations but rarely for research activities. “*Donor X made us swing to intervention rather than research.* […] *They want actions*” (R13). Secondly, the available funds had been planned and attributed well in advance, sometimes since several years: “*In the case of our research proposal, the donor was interested to finance the project, but it had to be aligned with the orientations of the institution. If the two don’t coincide, it won’t work. Even if the spokesperson of the identified donor is willing to collaborate, he or she cannot move things further*” (R7)*.*

Meeting with donors often led to adapting or replacing the initial proposal by another one for which budget was readily available. Similarly, some proposals were written by NetSRH members to respond to a local donor-initiated call after meeting with donors. Hence, they are not necessarily the South-led response to local needs.

#### Specific experience with networks and partnerships

Finding funds for research requires specific competences, namely one needs to be proactive and to have negotiation and persuasion capacities, as recognised by several NetSRH members: “*We have to make many more efforts to identify funds at national level that need to be consumed, for example within one of the ministries, and to identify academic institutions that may attract funds with whom we can partner. There is work to be done to see if we have explored all*” (R13)*.*

Some NetSRH members felt prejudged by potential northern partners: “*Maybe there are also unwritten elements that contribute to the fact that southern institutes don’t succeed in reaching a relatively important success rate. There is some kind of distrust from donors, especially external donors, concerning the southern institutes, in relation to their capacity. Perhaps the institutes of the south have not given sufficient guarantees*” (R6)*.* Meanwhile, when looking for partners, northern institutions will tend to gravitate towards the ‘better connected’ institutions, hence the increasing gap in experience between southern institutions: “*If you don’t have collaboration with institutions of the north, you never have the opportunity to be involved in a large-scale project for they don’t know you*” (R11)*.*

### Perceptions of NetSRH members on network collaboration between South and North

#### The appreciation of North–South collaboration

“*The ideal is to initiate our own research, for always answering to calls for proposals by northern institutes makes us an eternal second. As a consequence, we will never adequately learn how to present our own projects*” (R6)*.*

Southern NetSRH members considered the relation between northern and southern research partners asymmetric in (1) access to grants; (2) competences leading to such access (research skills but also research management skills); and (3) experience in networking and new communication technologies.

Several respondents reported a difference in access to grants between northern and southern institutions: “*Not all grants are open to the institutes of the south. When a northern institute partners with a southern institute, the latter is not always the beneficiary*” (R11)*.* North–South collaboration was compared with a ‘fair trade’ concept: “*Emphasis should be put on strengthening of the local capacity.* […] *Really trying to decentralize in a way that developing and European countries can truly be in partnership, with developing countries being the principal partner, the contractor. That gives the chance to southern institutions to receive overhead budget that may be used to strengthen local capacities*” (R11)*.*

NetSRH members presented the asymmetry as a cyclic problem: “*When the funds come from the north, perhaps the northern institutes are privileged due to their position: they know better how to present and are more aware of the capacities needed to obtain the results. They give more content, so they obtain more resources than institutes of the south*” (R6)*.*

Most NetSRH members envisioned a real discussion between northern and southern partners, on the distribution of tasks within the process of producing and publishing articles: “*We hope to be valued because we’re not only data collectors, but also share in the analysis and article writing. Things are improving now with partners of the north, who more and more organize postdoctoral stays. We find the funds, they invite us, and generally it is for one or two months and we write articles together. I think that is good*” (R15)*.* One respondent adds: “*In the north people are quite prompt in the production. The south needs to show some productivity as well, a certain aptitude to be able to rapidly produce the papers linked to the research conducted. I think it is through these measures that we will bring back the symmetry*” (R6)*.*

#### Intermediate challenges and successes of NetSRH

When asked whether NetSRH succeeded, at this stage of the project, in one of its major objectives, capacity strengthening, responses were mitigated. Building strong research institutions in the South remains challenging, as confirmed by respondents in our study, of whom some demonstrated a certain determinism: “*The asymmetry is there at the start, that is the problem. We cannot turn that into symmetry when we do not have the same competences*” (R6). When discussing the asymmetry in competences, respondents did not necessarily refer to a South–North dichotomy. Significant differences in competences between members exist despite, or precisely due to, an increase in multi-country projects in which more experienced southern research centres are selected as partners in large projects and are consequently more advantaged both financially and in terms of capacity strengthening. This also showed in the agenda of the annual meetings, which was made by NetSRH members in a consensual manner. While some NetSRH members wished to receive a refresher course concerning literature reviews, others were looking forward to technical sessions concerning ethics in research and more advanced training in qualitative research. The result was a meeting agenda with topics sometimes too advanced for certain members, yet not challenging enough to others.

#### Why common research projects between southern partners took time to develop

##### A perceived need for collaboration

Interestingly, not all southern network members felt, after 18 months of network activity, a true need to collaborate. Southern institutions collaborate little amongst themselves, confirmed NetSRH members: “*We are rather in competition than in partnership. We have the impression that every country had to propose a protocol and that the country that was in delay in comparison to others was ‘a less good student’*” (R9).

##### Communication

Poor communication among NetSRH members between annual meetings and the perception of competition by some members contributed negatively to the development of the South–South collaboration within NetSRH. Although there were perspectives for a potential research project in common, such collaboration has not yet taken place.

##### Financial perspectives

NetSRH not having a specific budget for conducting collaborative or joint research project was a limitation, and perceived so by the members.

“*Working with a cross-country partner who has no future, no budget whatsoever, and for whom it takes time and resources to be able to collaborate, does not ensure anything for thereafter. So people prefer to give the priority to what will give them a return the earliest possible*” (R11)*.*

##### Contextual differences

Another reason given by NetSRH members was the difference in epidemiological contexts of each country. The “*difficulty to find a unique donor or a transnational donor in every country*” (R8) is an additional constraint. There are also differences in professional culture; there had been some prejudices between North and West African NetSRH members in the beginning of the project.

#### What role do southern NetSRH research partners envision for their northern allies?

Although critical in their appreciation of capacity transfer through North–South networking, several respondents did consider partnerships with the North as important, sometimes even more important than investing in South–South networks. One respondent described two clear advantages: “*We need a cooperation with the north, not only to maintain a research quality standard which is relatively higher, but also to have the opportunity to access funds that are much more substantial and sustainable*” (R13).

With regards to having a southern instead of the traditional northern partner as the contracting party, hence entitled on overhead budget, a NetSRH member stated that:

“*This will not be evident. The institutions of the north will always try to keep the monopoly. It is not in their interest for southern institutions to become independent*” (R11).

While some respondents considered the coordination of a multi-country research project as a role for the ITM as sole northern partner, others attributed the weak collaboration between southern NetSRH partners precisely to the presence of a northern partner, and to the latter being the network lead: “*Perhaps people tend to turn to the ITM because it is the coordinating center*” (R6). However, as the same respondent adds: “*The northern partner coordinating the network does not justify a lack of direct collaboration between institutes of the south*” (R6).

According to NetSRH members, research capacity building should not only take place between North and South, yet increasingly between southern partners: “*It is important that with regards to contextualization of different questions socio-anthropologic aspects linked to our context, things are done through south-south collaboration or even at national level with other researchers*” (R13). Northern researchers do have a role to play as well though, for “*their vision on these questions is perhaps a bit more neutral than the vision of national researchers*” (R13).

## Discussion

Our study showed, half way through the network project, that individual coaching and collective capacity-building sessions as organised within NetSRH were appreciated by the members. The main constraints encountered in South-led research were partly linked to technical aspects of research but also to research management skills and experience, namely how to write a proposal which is appealing to donors, how to unblock funds and how to become the contracting party, entitled to overhead budget, instead of being the implementing partner. While the first two challenges are well known in the North as well, the last is an important one, and not easy to tackle. Hereto adding internet and communication constraints and a constant pressure to respond to calls for proposals in order to survive financially, South-led research becomes a somehow less important priority for NetSRH members. To respond to the request of the members, as expressed in this study, the focus of the last NetSRH meeting was on enhancing skills related to research management, grant writing, donor presentation and instruments to contribute to policy change.

### NetSRH: a North–South–South research network

The first of the 11 principles underlying North–South research partnerships, as developed by the Swiss Commission for Research Partnerships with Developing Countries as far back as 1998, is that partners should decide on objectives together [14]. NetSRH is financed by the Belgian Cooperation and its project proposal has been written by the only partner in the network not rooted in the south. Through more investment in collaboration between the southern partners, possibly to the detriment of the coaching role of the northern institute, the network might evolve towards South–South–North collaboration, which is one step away from the North–South network paradigm and one step closer to a reinforced South–South collaboration.

The United Nations Commission for Science and Technology for Development (UNCSTD) mentions, in its working paper Making North-South Research Networks Work, as a prerequisite to success the “*establishment of a strong common focus around a concrete, widely shared problem or goal*” [15]. Released in 1999, this report proves that the challenges experienced by NetSRH members are not new. Lessons from three African networks confirm this prerequisite [16]. Progress in the planned multi-country studies within NetSRH has been slowed down because of perceived differences in context, in research objectives and in focus, although it concerned the same theme.

A second recommendation found in the UNCSTD report and confirmed by the experiences of Doherty [16] is to achieve win-win situations and mutual benefits: “*Good network management implies both asking members for their voluntary inputs and helping them to benefit optimally from the network’s collective outputs*” [15]. Have we stretched the voluntary inputs of the network too far? Did the coaching, capacity building and opportunities for exchange in the network constitute a return on the investment that the members have made? When asked for possible reasons of the lack of sharing and collaboration between the network members of the south, the answer was often reduced to logistical and pragmatic reasons: a lack of time, a deficient internet connection and weak communication modalities in general, including differences in context.

NetSRH has gone far beyond most recommendations for North–South networks [15]: a formal network governance structure exists, southern researchers’ participation is optimised and NetSRH members are encouraged to participate actively by informing the network of the results of their research activities and other news, such as technical information or details concerning calls for proposals in fields of common interest. Nevertheless, from our study results collected after 18 months of launching the NetSRH, we derive the observation that southern partners perceive a certain domination of the north over the south. NetSRH members attribute this phenomenon largely to the fact that such networks, as well as research projects developed within those networks, are mostly initiated and financed by northern partners. This maintains LMIC in an asymmetric relation with their research partners from the north [17]. Is this perception correct?

Weinrib [18] argues that the asymmetry between northern and southern research partners is rooted in (distorted) program and project selection criteria and processes, in project agenda-setting processes, in knowledge management and resource management as well as in a predominance of short-term funding cycles and the limited success of institutionalisation efforts of North–South collaboration. Weinrib documents how the initiation of North–South research collaboration has historically been based on exogenous political and academic agendas rather than endogenous motives in order to maintain a high level of dependency within the south on foreign knowledge-related norms, structures and systems. Hereby southern researchers “*remain beholden to particular mandates of knowledge production and evaluation vis-à-vis supply-side development and knowledge regimes*” while northern actors “*continue to place themselves at the center of the knowledge management systems, often as gatekeepers of technical knowledge and base practices*” ([15] p. 103). Interviews with NetSRH members confirm that northern donors have funding available only for particular fields of interests and that southern institutes will adapt their production to that reality in the absence of alternative funding sources.

When research takes place in the South, southern partners feel they are predominantly responsible for more executive tasks, e.g. data collection, rather than for analytic, technical or coordinating aspects of a given research project, e.g. dissemination of results or production of articles. We might question whether this domination of North over South is unique; whether there is not also a domination by southern partners with more experience in networks over other southern partners? As we have seen in our study, significant differences exist in research and other technical competences among NetSRH partners, as well as in the experience gathered in multi-country projects and in working with foreign donors. Are these differences solely a consequence of an unequal distribution of resources, competences and exposure to experience? Is it hence a matter of closing the gap through “tailor-made” capacity building in order to make those who need to progress faster? Bennet et al. [19] showed that institutes that benefitted from longer-term institutional collaborations with organisations outside their own country were particularly advantaged concerning capacity development. Similarly, Mayhew et al. [3] confirmed that pre-existing research capacity, as well as good personal relationships between members of the partner institutions, increased the likelihood of successful partnership. In the experience of Mayhew et al. [3], the northern partner’s biggest lesson was how to manage the very different types of partners within the same partnership. It requires space and flexibility for the different partners in order to define their own national priorities while engaging each partner in international research interests.

Is asymmetric capacity building with a focus on those who are lagging behind potentially key to progressively increasing the efficacy of South–South collaboration? In other words, when trying to close the knowledge gap, should we move from North–South collaboration to South–South collaboration? Or is the domination of one group over the other associated to directing political, economic and often historically embedded power relations? Would it be possible for the stronger southern partners to train the other southern partners without maintaining (or even reinforcing) the asymmetry in their relationship? Is a transfer of competence and knowledge by the stronger southern partners to less experienced peers a better response than the traditional North–South capacity building, or would that only mean jumping from the frying pan into the fire?

Interesting, and potentially a key to progress, is the observation by Bennett et al. [19] that the more successful institutes appeared to benefit from strong links to policymakers which affected not only their ability to influence policy, but also supported their research capacity. A similar observation has been made in the NetSRH experience.

### Why to invest in networks?

In the wake of the questions discussed above, we ask ourselves whether it is worth investing in networks and what the role of the northern partner should be in such networks. If research development in the South is the main objective, the institutional collaboration run by the ITM over the last decade in 10 to 15 countries has shown more efficiency. The network is not a guarantee of collaboration. However, it does create a pool of researchers who can react more rapidly when an opportunity occurs to collaborate in projects. In this sense and with South–South collaboration being an objective, networking may lead to several advantages, despite it being a time and resource-consuming activity. These advantages become apparent after a certain investment, as we see in the NetSRH project in which members have started to collaborate spontaneously only 2 years after its implementation.

### Lifting the barriers to South-led research: heading to true and equal partnership

Our study has exemplified a vicious circle. Researchers in the South run after consultancies to secure income, yet large budgeted projects are usually North-led, whereby southern institutions are asked to collect the data according to a study design decided upon by the northern partner. Consequently, southern partners do not have time to invest in reading, writing and submitting their own projects. When there is no project (no income), the researchers seek consultancies to make ends meet without motivation to invest in things they do not master, hence capacity strengthening is put on the back burner. Much of the work undertaken by the institutes appears to have been driven by requests from government or donors [20]. Girvan argues that, if governments and donors are not prepared to provide resources in a form of assistance which is non-intrusive and supportive of the development of local capacities, it should not be offered, and if offered it should not be accepted, for otherwise it will hinder development through continuous knowledge dependency and disempowerment [7]; this is easier said than done. As long as research centres in LMICs are struggling to find structural financing and hence unable to advance the costs for literature reviews and other preparative studies for autonomously led research, it could be suicidal for centres to refuse offers that are not as supportive as they need to be. It is evidently a shared responsibility between partners, yet the donor agencies should be urged to collaborate with their partners on an equitable and ethical offer for assistance, ensuring a fair share for its partners in overheads, task distribution, output and authorship. Donors must commit to long-term and in-country support if they wish to support successful research capacity building [3].

LMICs might take a prominent role in leading or directing these research collaborations in order to maximise the benefits and minimise the harm of inherently inequitable relationships. Chu et al. [5] suggest that high-income country (HIC) institutions could provide access to distance learning resources such as online libraries, proposal development, statistical expertise, database development and management, and that trusted long term HIC collaborators who understand the context and needs of the region can teach agenda-setting skills and assist in agenda development. Further, they also state that the coordination of HIC collaborators should be done by the LMIC. The ITM is doing so since 1998 through its institutional collaboration in 14 countries for which they are financially supported by the Belgian Development Cooperation. South-led research has also been stimulated by China and South Africa in, respectively, the (rather contested^2^) 1000 Talent Plan for foreigners [21] and in the South African Research Chairs Initiative [22], aimed at recruiting or maintaining internationally recognised academic experts in order to strengthen and improve the country’s research and innovation capacities.

Finally, while collaborative partnership initiatives have rightfully received a growing interest and are the main topic of our study, we also wish to stress the importance of a fair distribution of authorship credit within these partnerships, which needs similar attention [23].

### Recommendations

Mid-term of the network project, NetSRH members from South and North unanimously conclude that sustainable financing of southern research centres is a necessary condition for them to initiate their own research projects. We recommend reserving funds within the international donor agencies for South-led research in order to break the vicious circle of running behind money provided by northern donors, thereby missing out on time and resources for literature review and needs evaluations required for initiating own research. Reserving the necessary funds can be done in at least two ways: international donors can finance studies decided by the southern partners through the demand from Ministries of Health (which is itself financed by international donors) or they can allocate a part of the international funds to South-led research only.

With regards to competences, inequality between network partners could represent an opportunity rather than a source of inequity: the more advanced southern partners discussing with and informally coaching their less experienced peers appeared, at least for NetSRH, to be a very efficient way to strengthen the competences of all. Moreover, doing so in the institute of one of the southern partners reduces geographical boundaries and reinforces the organisational capacities and experience of the hosting partner.

Other important assets for successful North–South partnering, equally highlighted by Doherty in 2015 [16], are a strong anchoring institution with the financial and human resources to sustain the network/collaboration, dedicated resources for collaboration in terms of funding and regular face-to-face meetings.

## Conclusion

NetSRH, a research network gathering mainly southern research partners, has been considered a relatively efficient tool for capacity transfer by its members. However, the capacity to generate and run research projects autonomously does not come over a day. The current donor architecture seems to keep southern research partners dependent. In order to remain viable, southern researchers work on research projects that are financially rewarding, rather than on those that they believe respond to the country’s highest needs. Therefore, the research agenda is still indirectly set by foreign donors and in-country offices of UN agencies. A mixed South–South–North network, as NetSRH, is a step in the good direction, but not a guarantee for collaboration. Moreover, within NetSRH concrete south-south collaboration remained limited in the first half of the project. Reasons given were pragmatic: no budget, no time, deficient communication means, and other priorities. The main limitation of the NetSRH project is that there is no fund for research readily available. The ITM coaches its partners in the search for funds, among others through training in grant writing and policy briefs. Still, as we have seen in the second half of the NetSRH project, a network has the potential to considerably enhance the partners’ reactivity, since a pool of various expertise is created, turning time initially spent in network activities into time won at the moment the partners wish to apply for large grants.

NetSRH members unanimously concluded that sustainable financing of southern research centres is a necessary condition for them to initiate their own research projects. We recommend reserving funds within the international donor agencies for South-led research in order to break the vicious circle of running behind money provided by northern donors, thereby missing out on time and resources for reviewing research gaps and/or conducting needs evaluations required to initiate relevant own research.

## Footnotes

1 The NetSRH members are: Agence de Formation, de Recherche et de l’Expertise en Santé pour l’Afrique (AFRICSanté, Burkina Faso); Centre régional de Formation, Recherche et de Plaidoyer en santé de la reproduction (CEFOREP, Senegal); Centre de Recherche en Reproduction Humaine et en Démographie (CERRHUD, Benin); Ecole Nationale Supérieure de Statistique et d’Economie Appliquée (ENSEA, Côte d’Ivoire); Ecole Nationale de Santé Publique (Morocco); Délégation Régionale de l’Office Nationale de la Famille et de la Population (ONFP) de Nabeul (Tunisia) et Direction Régionale de la Santé (DRS) de Nabeul (Tunisia); Centre National de Formation et de Recherche en Santé Rurale de Maferinyah (CNFRSR Maferinyah, Guinea); Université Badji Mokhtar d’Annaba (Algeria).

2
www.scmp.com/news/china/article/1631317/chinas-programme-recruiting-foreign-scientists-comes-under-scrutiny
